# Germline and somatic variations influence the somatic mutational signatures of esophageal squamous cell carcinomas in a Chinese population

**DOI:** 10.1186/s12864-018-4906-4

**Published:** 2018-07-16

**Authors:** Jintao Guo, Jiankun Huang, Ying Zhou, Yulin Zhou, Liying Yu, Huili Li, Lingyun Hou, Liuwei Zhu, Dandan Ge, Yuanyuan Zeng, Bayasi Guleng, Qiyuan Li

**Affiliations:** 1 Department of Translational Medicine, Medical College of Xiamen University, Xiamen, 361102 China; 2 Center for BioMedical Big Data Research, Medical College of Xiamen University, Xiamen, 361102 China; 3 Central Laboratory, Zhongshan Hospital affiliated to Xiamen University, Xiamen, 361004 China; 4 Maternity and Child Health Care Hospital, Xiamen, 361003 China; 5 Department of Pediatrics, The First Affiliated Hospital of Xiamen University, Xiamen, 361003 China; 6 Department of Gastroenterology, Zhongshan Hospital affiliated to Xiamen University, 201 Hu’bin South Road, Xiamen, Fujian Province China; 7 Medical College of Xiamen University, 4221-120 South Xiang’an Road, Xiang’an District, Xiamen, Fujian Province China

**Keywords:** Esophageal squamous cell carcinomas, *ZNF750*, *CDC27*, Mutational signature, Genetic burden

## Abstract

**Background:**

Esophageal squamous cell carcinomas (ESCC) is the fourth most lethal cancer in China. Previous studies reveal several highly conserved mutational processes in ESCC. However, it remains unclear what are the true regulators of the mutational processes.

**Results:**

We analyzed the somatic mutational signatures in 302 paired whole-exome sequencing data of ESCC in a Chinese population for potential regulators of the mutational processes. We identified three conserved subtypes based on the mutational signatures with significantly different clinical outcomes. Our results show that patients of different subpopulations of Chinese differ significantly in the activity of the “NpCpG” signature (FDR = 0.00188). In addition, we report *ZNF750* and *CDC27*, of which the somatic statuses and the genetic burdens consistently influence the activities of specific mutational signatures in ESCC: the somatic *ZNF750* status is associated with the AID/APOBEC-related mutational process (FDR = 0.0637); the somatic *CDC27* copy-number is associated with the “NpCpG” (FDR = 0.00615) and the AID/APOBEC-related mutational processes (FDR = 8.69 × 10^− 4^). The burdens of germline variants in the two genes also significantly influence the activities of the same somatic mutational signatures (FDR < 0.1).

**Conclusions:**

We report multiple factors that influence the mutational processes in ESCC including: the subpopulations of Chinese; the germline and somatic statuses of *ZNF750* and *CDC27* and exposure to alcohol and tobacco. Our findings based on the evidences from both germline and somatic levels reveal potential genetic regulators of the somatic mutational processes and provide insights into the biology of esophageal carcinogenesis.

**Electronic supplementary material:**

The online version of this article (10.1186/s12864-018-4906-4) contains supplementary material, which is available to authorized users.

## Background

Esophageal cancer (EC) is the second most common gastrointestinal cancer worldwide. EC causes 400,000 deaths annually, which accounts for 4.9% of total deaths from cancer [1]. EC consists of two major histological types, esophageal adenocarcinoma (EAC) and esophageal squamous cell carcinoma (ESCC). Each disease is characterized by distinct pathological and genomic features [2]. The incidence rates of EAC and ESCC differ significantly among different regions. The burden of EAC incidence is higher in Northern and Western Europe [3], while the burden of ESCC incidence is higher in South-Eastern and Central Asia (79%). 53% of global cases of ESCC (210,000 cases per year) are diagnosed in China, which makes it the fourth most lethal cancer in the country. The difference in the incidence rates is attributed to diverse epidemiological and genetic factors [4].

Cancers accumulate various somatic alternations in the genome. The somatic landscape of the tumor genome is a result of different mutational processes that occur throughout the course of the disease [5–7]. The mutational processes in cancers are driven by various determinants, the microenvironment, risk factors and genetic variations. The biological background of the mutational processes in diverse cancer types have been revealed by recent studies, such as APOBEC-induced deamination, deamination of methylated cytosine, tobacco and alcohol exposure and so on [8–10]. The mutational processes are represented by distinct patterns of frequencies of trinucleotide sequences surrounding the base of substitution, which is also known as the somatic mutational signatures [11]. The relative activities of the mutational signatures surrogate for the rate of the corresponding mutational processes. Many studies derive algorithms to detect mutational signatures from genome or exome sequences of cancer [12–14]. These algorithms are usually based on decomposition of the mutational profiles. The accuracy and power of the derivation are subject to the complexity of the model as well as the sample size. Recent methods, such as the “probabilistic mutation signature”, improve the reliability of the discovery of the mutational signatures from small sample by reducing the number of parameters [13, 15].

Notably, more and more studies show evidences that the somatic mutational processes in cancers are associated with the somatic or germline statuses of certain genes [12, 16]. For instance, the overall somatic mutational burden in melanoma is associated with germline *MC1R* status [17]; and in breast cancer it is associated with the germline *RAD51B* status (rs2588809) [18]. At the somatic level, the activity of the signature 5 in the Catalogue Of Somatic Mutations In Cancer (COSMIC) is associated with the somatic *ERCC2* status in urothelial tumors [19]; and the activity of the APOBEC-related mutational signature in ESCC is associated with the somatic *PIK3CA* and *ZNF750* statuses [20, 21]; Plus, specific mutational signatures are used to predict *BRCA1* and *BRCA2* deficiency in breast cancer [22]. These studies suggest that both germline and somatic alterations can drive the mutational processes in cancers and the driver genes may play a decisive role in the development of the disease.

In this study, we used paired whole-exome sequencing (WES) data to identify the regulators of the somatic mutational processes in ESCC in a Chinese population by combining evidences from both germline and somatic levels [20, 23–25]. We assessed the association between the activities of the somatic mutational processes and various gene sets including the significantly mutated genes (SMGs), the genes with somatic copy-number alterations (SCNAs) as well as the GWAS risk loci of ESCC. We considered the effects from the germline and somatic levels to suggest several potential determinants of the somatic mutational processes; and provide insights into esophageal carcinogenesis and inform the future diagnosis and therapy.

## Results

### Somatic mutational signatures

**Fig. 1:**
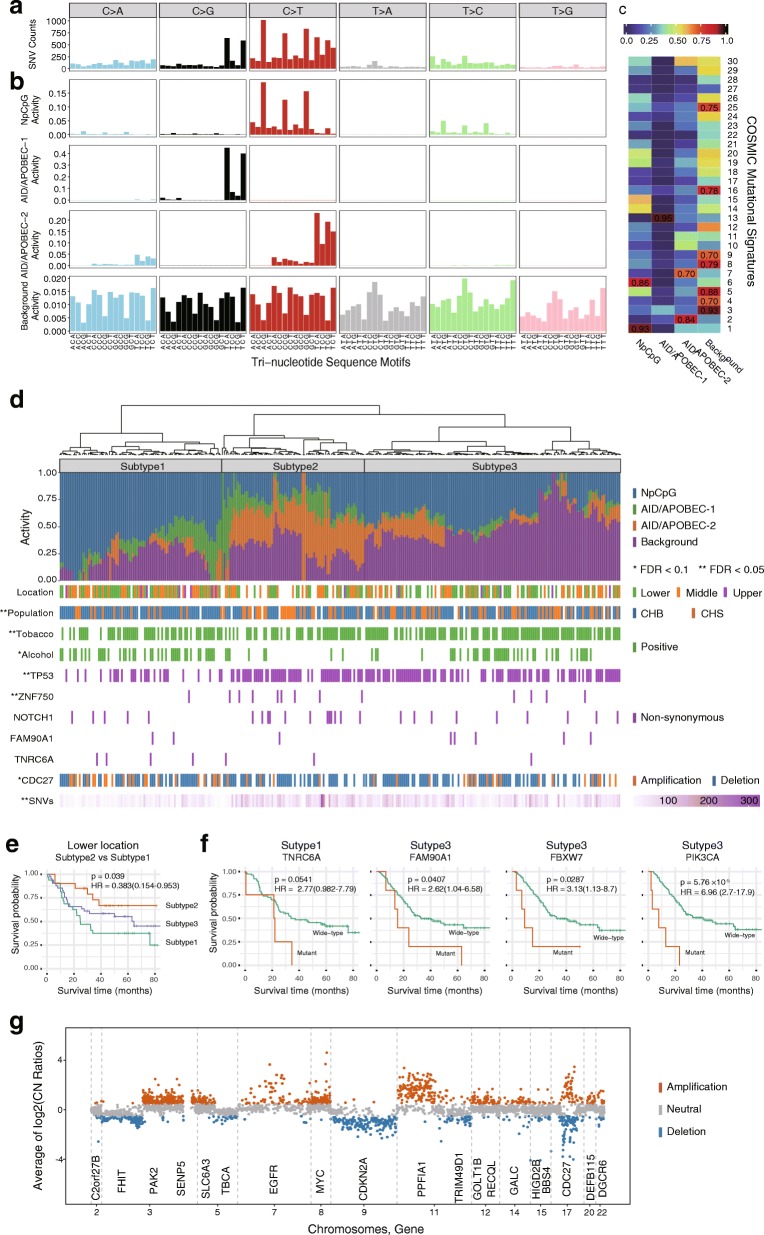
Somatic mutational signatures in 302 ESCC. **a** Frequencies of 96 subtypes of base substitutions of SNV identified from the exomes of 302 ESCC. **b** Four somatic mutational signatures, “NpCpG”, “AID/APOBEC-1”, “AID/APOBEC-2” and “Background” retrieved from the mutational profiles of SNVs. **c** Comparison of the four somatic mutational signatures from ESCC with the known mutational signatures (No.1 to 30) in the COSMIC. The similarity measures labeled in the grids are based on “Cosine similarity”. **d** Subtypes of ESCC based on the clustering of the activities of the mutational signatures of the ESCC samples. The activities of each mutational signatures in each sample are denoted by the colored-bars below the dendrogram. The clinical and molecular features that are significantly associated with the subtypes are labeled beneath the bar plot. **e** The outcome of the three subtypes of ESCC differ in tumors in lower esophagus. The Kaplan-Meier curves are based on the fraction of overall survival in lower esophagus. The *P* values and Hazard Ratio (95% confidence interval) are estimated using Cox-regression. **f** The SMGs are predictive in subtype 1 and 3. Kaplan Meier curves are based on the somatic *TNRC6A*, *FAM90A1*, *FBXW7* and *PIK3CA* mutational statuses. The *P* values and Hazard Ratio (95% confidence interval) are estimated using Cox-regression. **g** Log2 copy-number ratio of the genomic segments in ESCC. The segments of significant copy number alterations are shown in color (orange: amplification, blue: deletion) with the affected genes labeled beneath. A copy-number amplification was defined if the log2 copy number ratio is above 0.5; and the deletion if the value is below − 0.5

We identified 13,854 single nucleotide variants (SNVs) and 2274 insertions and deletions (InDels) from 302 paired exonic sequences of ESCC. The median rate of the mutations is 1.11 per megabase (range 0.03–9.21; Additional file 1: Table S1 and Additional file 2: Figure S1). Of all the SNVs and InDels, 73.5% (7331) are non-synonymous variants; 53.2% (8586) are annotated in the COSMIC v79 [26]. The six subtypes of base substitutions (C > A, C > G, C > T, T > A, T > C and T > G) are unevenly represented in the SNVs. C > T is the most common substitution in ESCC (6391, 39.6%), followed by C > G (2,234, 13.9%; Fig. 1a).

We retrieved four highly conserved somatic mutational signatures from the mutational profiles (Fig. 1b). The signatures are highly comparable to the known ones in the COSMIC database based on the cosine similarity (CS; Fig. 1c) [16, 26]. The “NpCpG” signature (characterized by C > T at NpCpG trinucleotide), which has been previously reported in ESCC, is highly similar to the COSMIC signature 1 (deamination of 5-methycytosine; CS = 0.93) and signature 6 (defective DNA mismatch repair; CS = 0.86) [20]. The “AID/APOBEC-1” signature (C > G at TpCpN trinucleotide) and “AID/APOBEC-2” signature (C > T at TpCpN trinucleotide) match the COSMIC signature 13 (CS = 0.95) and signature 2 (CS = 0.84), respectively. Both signatures are driven by AID/APOBEC family of cytidine deaminases activity [16, 26]. Moreover, “AID/APOBEC-2” signature is associated with COSMIC signature 7 (CS = 0.70), which presents in multiple squamous cancers. Finally, the “Background” signature represents the base level mutational processes in ESCC which correlates strongly to COSMIC signature 3 (the failure of double-strand-break repair by homologous recombination; CS = 0.93), signature 4 (tobacco exposure; CS = 0.70), signature 9 (the activity of AID during somatic hypermutation; CS = 0.70) and several other signatures of unknown background (COMIC signature 5, 8, 16, 25) [13].

We identified three conserved subtypes of ESCC based on the relative activities of the mutational signatures. Subtype 1 (*n* = 85) is characterized by the “NpCpG” signature activity and the relatively low burden of somatic SNVs (FDR = 3.85 × 10^− 9^; Fig. 1d). *CDC27* somatic amplification is significantly enriched in this subtype (*n* = 22, FDR = 0.0682). Subtype 2 (*n* = 75) is characterized by the “AID/APOBEC-2” signature activity and patients who do not drink alcohol. The non-synonymous mutations of *ZNF750* (*n* = 8, FDR = 0.0471; Fig. 1d) are overrepresented in this subtype. Finally, subtype 3 (*n* = 134) is characterized by the “Background” signature and smoking patients (*n* = 103, FDR = 0.0169; Fig. 1d). Besides, subtype 3 also significantly enriches *TP53* mutations (*n* = 89, FDR = 7.38 × 10^− 7^; Fig. 1d).

The clinical outcomes of the three subtypes are also different. In the lower esophagus, subtype 2 show significantly higher overall survival rate than subtype 1 (HR = 0.383, 95% CI = 0.154–0.953, *P* = 0.039; Fig. 1e).

### Significantly altered genes in ESCC

We identified 12 SMGs (FDR < 0.1; Additional file 2: Figure S1). We compared the SMGs to known cancer genes annotated in the COSMIC as well as other published studies, most of the SMGs we identified (*TP53*, *NOTCH1*, *FAT1*, *ZNF750*, *RB1*, *PTCH1*, *PIK3CA*, *FBXW7*, *NFE2L2* and *CDKN2A*) are consistent to the ones reported previously in ESCC [20, 23–25]. Our results suggest two new candidate genes: *FAM90A1* (family with sequence similarity 90, member A1, MIM: 613041, FDR < 1 × 10^− 10^) and *TNRC6A* (trinucleotide repeat containing 6A, MIM: 610739, FDR = 9 × 10^− 10^). For *FAM90A1*, we report an in-frame insertion (c.1031_1032insCGT [p.T344_S345insV]), which presents in 8 patients. And for *TNRC6A*, we report an in-frame deletion (c.333_344del12 [p.P115_Q118delPQPQ]) which presents in seven patients (Additional file 3: Table S2). It’s also noteworthy that both InDels are previously annotated in other TCGA cancer cohorts but not in ESCC [27–29].

In addition, the SMGs are selectively predictive in the subtypes of ESCC defined by the mutational signatures. For example, *TNRC6A* mutation is suggestively associated with poor overall survival in subtype 1 (HR = 2.77, 95% CI = 0.982–7.79, *P* = 0.0541); whereas the somatic statuses of *FAM90A1* (HR = 2.62, 95% CI = 1.04–5.58, *P* = 0.0407), *FBXW7* (HR = 3.13, 95% CI = 1.13–8.7, *P* = 0.0281) and *PIK3CA* (HR = 6.96, 95% CI = 2.7–17.9, *P* = 5.76 × 10^− 5^) are significantly predictive in subtype 3 (Fig. 1f).

On a different note, we identified 76 regions of significant somatic copy number alterations (SCNAs, FDR < 0.01; Additional file 4: Table S3) in ESCC, of which 39 are amplifications; 37 are deletions/losses. These regions include not only known SCNA events in ESCC (7 amplifications and 23 deletions) and ECA (6 amplifications and 6 deletions) [20, 24, 25, 30–33], but also 40 new events such as deletions in 5q14.1 (57.6%) and 17q21.32 (50.0%), amplifications in 10q11.21 (43.0%) and 17p11.1 (40.4%). We retrieved 20 genes located within the regions of the SCNAs, such as *CDKN2A* (9p21.3; FDR = 3.76 × 10^− 92^), *MYC* (8q24.21; FDR = 9.34 × 10^− 54^) and *FHIT* (3p14.2; FDR = 5.28 × 10^− 28^). We noticed that *CDC27* (17q21.32) is both amplified in 15.90% (*n* = 48; FDR = 2.68 × 10^− 10^) and deleted in 51.6% (*n* = 156; FDR = 9.05 × 10^− 22^) of the patients (Fig. 1g; Additional file 5: Table S4).

We retrieved the protein-protein interaction (PPI) networks for both the SMGs (MutSigCV *p* < 0.05) and the SCNA-related genes, from which we identified 4 subnetworks undergoing significant somatic modification (*p* < 0.05; Additional files 2 and 6: Figures S1 and S2). The subnetworks significantly enrich for the KEGG pathways of cell cycle (FDR = 2.03 × 10^− 9^), P53 signaling (FDR = 4.10 × 10^− 5^), NOTCH signaling (FDR = 0.00163) and many other tumor related pathways (Additional file 7: Table S5a). Plus, the genes in the subnetworks significantly overrepresent DNA motifs of known transcription factor binding sites such as CEBPD (FDR = 0.00991), CEBPA (FDR = 0.00991) and many others (Additional file 7: Table S5b).

Finally, we report eight rarely mutated genes (mutational frequency < 2%), including *CUL3*, *PTEN*, *RBPJ*, *EIF2S2*, *WAC*, *ANAPC10*, *CD7* and *S100A2* (Additional files 2 and 6: Figures S1 and S2).

### Risk factors associated with somatic mutational processes in ESCC

**Fig. 2:**
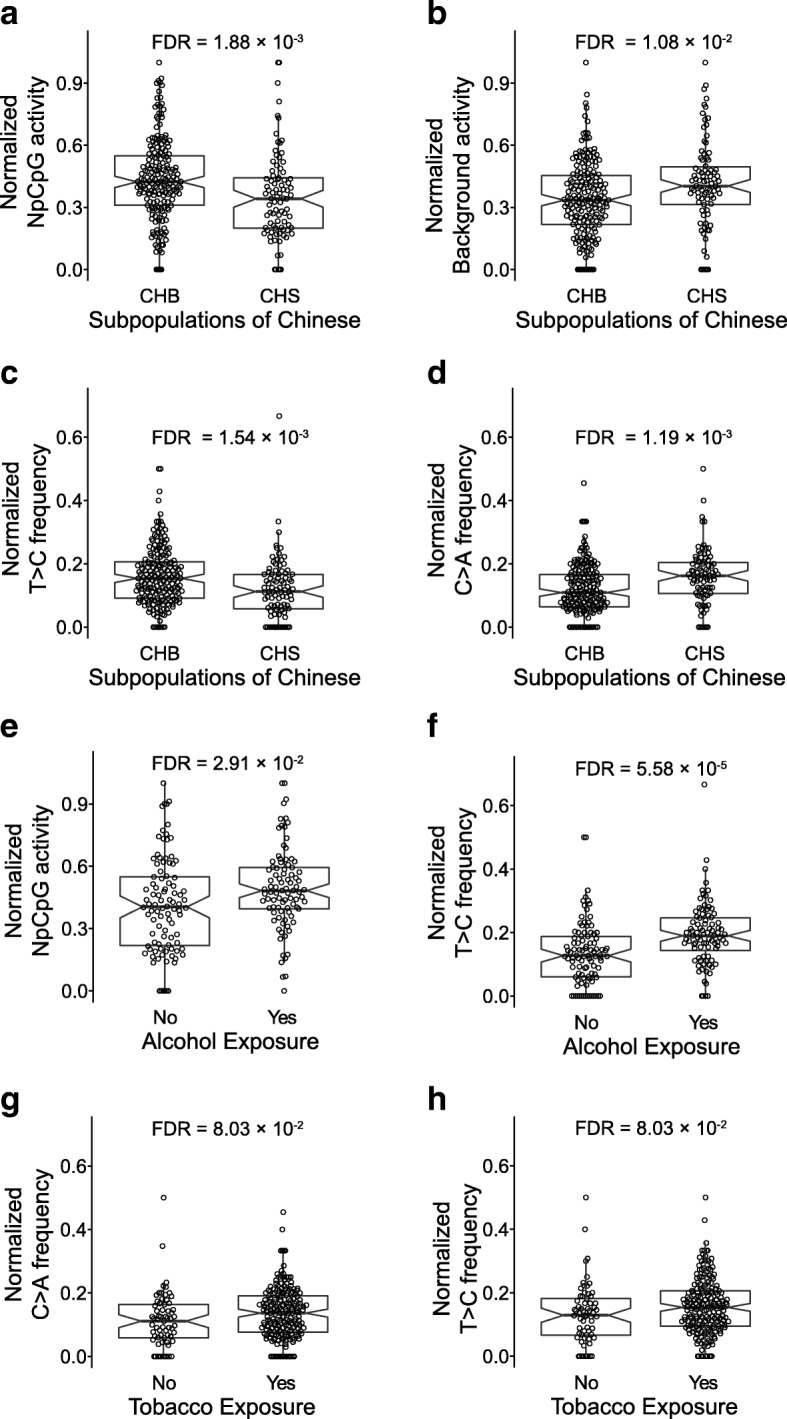
The mutational processes in ESCC are associated with subpopulations of Chinese, tobacco and alcohol exposure. Comparison of the activities of the “NpCpG” signature (**a**), the “Background” signature (**b**) and the frequency of C > A (**c**) and T > C (**d**) substitutions in CHBs and CHSs. The subpopulations are determined based on the reference populations of CHS and CHB from the TGP. Comparison of the activities of the “NpCpG” mutational signatures (**e**) and the frequencies of T > C substitutions (**f**) against the alcohol exposure. And comparison of the frequencies of C > A substitutions (**g**) and the frequencies of T > C substitutions (**h**) with tobacco exposure. The FDR is based on the adjusted Wilcoxon rank-sum test *P* values

Our results show that the activity of “NpCpG” signature is significantly higher in the subpopulation of “Han Chinese in Beijing, China” (CHB) than those from the “Han Chinese South” (CHS; FDR = 0.00188); the activity of the “Background” signature is significantly higher in CHS than in CHB (FDR = 0.0108; Fig. 2a and b, Additional file 8: Figure S3). As for specific subtypes of substitutions, the frequency of C > A is higher in CHS (FDR = 0.00119) whereas the T > C frequency is higher in CHB (FDR = 0.00154; Fig. 2c and d). These results suggest that the population-genetical background strongly influences somatic mutational processes in ESCC.

We further assessed the association between other risk factors and the activity of the mutational signatures. As results, exposure to alcohol is strongly associated with higher frequency of T > C substitution (FDR = 5.58 × 10^− 5^) as well as the activity of the “NpCpG” signature (FDR = 0.0291; Fig. 2e and f). On the other hand, exposure to tobacco moderately associates with higher frequency of C > A (FDR = 0.0803) and T > C substitution (FDR = 0.0803; Fig. 2g and h).

### Genetic regulators of the mutational signatures in ESCC

The identification of the driver genes is key to understand the mutational processes in cancers. Here we evaluated the gene sets aforementioned in ESCC for the effects on the mutational processes.

### Somatic level

**Fig. 3:**
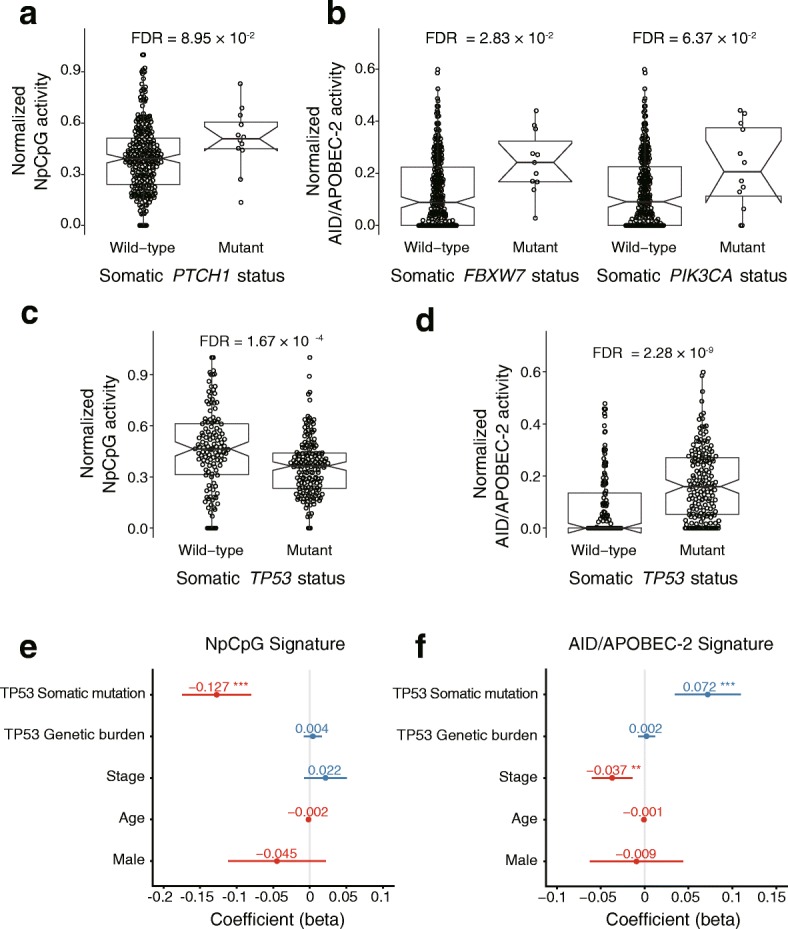
The mutational processes are associated with the somatic status of the SMGs. The activity of the “NpCpG” signature is associated with the somatic statuses of *PTHC1* (**a**). The activity of the “AID/APOBEC-2” signature is associated with the somatic statuses of *FBXW7* and *PIK3CA* (**b**). The somatic statuses of *TP53* is associated with the activity of the “NpCpG” signature (**c**) and the “AID/APOBEC-2” signature (**d**). The FDR is based on the adjusted Wilcoxon rank-sum test *P* values. The effects for the somatic status and the genetic burden of *TP53* on the activity of the “NpCpG” signature (**e**) and the “AID/APOBEC-2” signature (**f**) are shown with other clinical features. The *P* values are based on multivariate regression analysis: *P < 0.05, **P < 0.01, ***P < 0.001

**Fig. 4:**
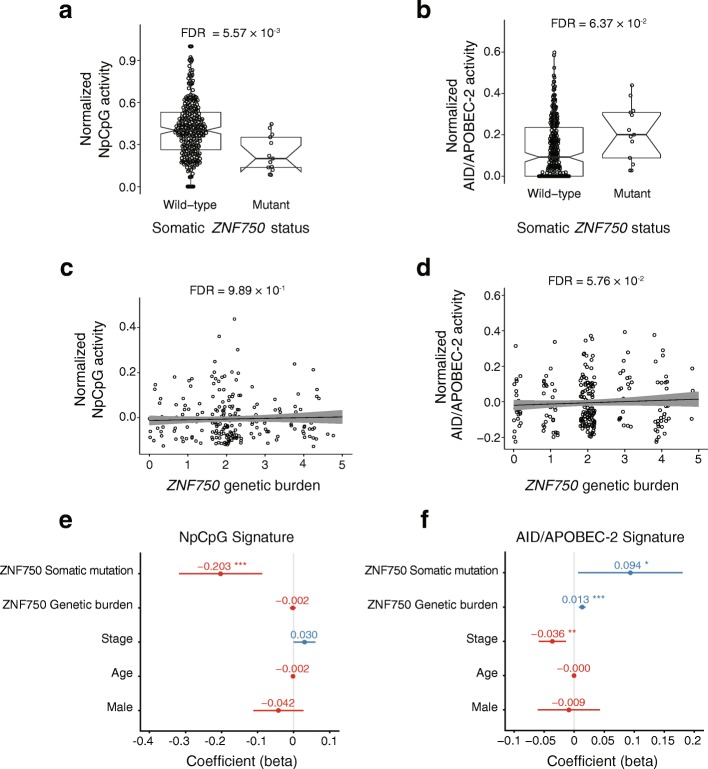
The mutational processes are associated with the somatic status and the genetic burden of *ZNF750*. The activity of the “NpCpG” (**a**) and “AID/APOBEC-2” signature (**b**) is associated with the somatic *ZNF750* statuses. The FDR is based on the adjusted Wilcoxon rank-sum test *P* values. The activity of the “NpCpG” (**c**) and “AID/APOBEC-2” signature (**d**) is associated with the genetic burden of *ZNF750*. The FDR is based on the adjusted SKAT *P* values, with the effects of the age, the clinical stage and ancestry being corrected. The effects of the somatic status and the genetic burden of *ZNF750* on the activity of the “NpCpG” signature (**e**) and the “AID/APOBEC-2” signature (**f**) are shown with other clinical features. The *P* values are based on multivariate linear regression analysis: *P < 0.05, **P < 0.01, ***P < 0.001

We find that the activity of the “NpCpG” signature increases in tumors carrying non-synonymous somatic *PTCH1* mutations (FDR = 0.0895; Fig. 3a); but decreases in tumors with non-synonymous somatic mutations in *TP53* (FDR = 1.67 × 10^− 4^; Fig. 3c) and *ZNF750* (FDR = 0.00557; Fig. 4a). Moreover, the activity of the “AID/APOBEC-2” signature activity significantly increases with the somatic mutations of *FBXW7* (FDR = 0.0283; Fig. 3b), *PIK3CA* (FDR = 0.0637; Fig. 3b), *TP53* (FDR = 2.28 × 10^− 9^; Fig. 3d) and *ZNF750* (FDR = 0.0637; Fig. 4b). When it comes to specific subtypes of substitutions, the mutational statuses of 5 SMGs (*ZNF750, TP53, FAT1*, *FBXW7* and *PIK3CA*) are significantly associated with the increased overall burden of SNVs (FDR < 0.1; Additional file 9: Figure S4a). In particular, the somatic *TP53* status is significantly correlated with high frequency of C > A, C > T, T > A and low frequency of T > C substitutions (FDR < 0.1; Additional file 9: Figure S4b).

**Fig. 5:**
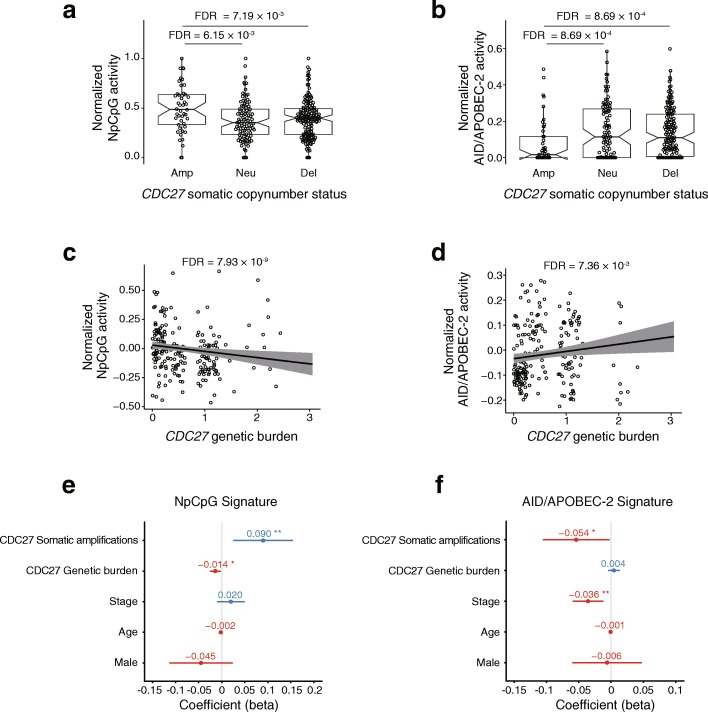
The mutational processes are associated with somatic copy number status and genetic burden of *CDC27*. The activity of the “NpCpG” (**a**) and “AID/APOBEC-2” (**b**) signature is associated with the somatic copy number amplification of *CDC27*. The FDR is based on the adjusted Wilcoxon rank-sum test *P* values. The activity of the “NpCpG” (**c**) and “AID/APOBEC-2” (**d**) signature is associated with the genetic burden of *CDC27*. The FDR is based on adjusted SKAT *P* values, with the effects of the age, the clinical stage and ancestry being corrected. The effects of the somatic copy number amplification and the genetic burden of *CDC27* on the activity of the “NpCpG” signature (**e**) and the “AID/APOBEC-2” signature (**f**) are shown with other clinical features. The *P* values are based on multivariate linear regression analysis: *P < 0.05, **P < 0.01, ***P < 0.001

Genes undergoing somatic copy number alterations can also influence the mutational processes in ESCC. For instance, *MYC* amplifications, *CDC27* deletions and *FHIT* deletions are all significantly associated with the higher burden of SNVs (FDR < 0.1; Additional file 9: Figure S4c). And the *CDC27* amplifications, alone, is associated with lower frequency of C > A substitution (FDR = 0.00216; Additional file 9: Figure S4d). As for the mutational signatures, the activity of the “NpCpG” signature increases significantly with somatic *CDC27* amplifications (FDR = 0.00615; Fig. 5a) while the activity of the “AID/APOBEC-2” signature decreases with the same event (FDR = 8.69 × 10^− 4^; Fig. 5b).

### Germline level

For the genes that influence the mutational processes somatically, we further interrogate if the burden of germline polymorphisms shows consistent effect. As results, the genetic burdens of *ZNF750* (FDR = 0.0576; Fig. 4d) and *PTCH1* (FDR = 0.00818; Additional file 10: Figure S5a) are significantly associated with increased activity of the “AID/APOBEC-2” signature. And the genetic burden of *CDC27* is associated with both the activity of the “NpCpG” signature (FDR = 7.93 × 10^− 9^; Fig. 5c) and the “AID/APOBEC-2” signature (FDR = 0.00736; Fig. 5d). In addition, our data show that the genetic burdens of *NOTCH1*, *TP53*, *PTCH1* and *CDC27* are significantly associated with either the overall burden of SNVs or specific subtypes of substitutions (Additional files 10 and 11: Figures S5b, c and S6a). These findings suggest that *ZNF750* and *CDC27* are major candidate driver genes of the mutational processes in ESCC.

We investigated another 56 genes which are related to the known GWAS risk loci of ESCC in Chinese population. As results, four genes (*DNAH11*, *CHEK2*, *HECTD4* and *HEATR3*) are associated with the activities of either mutation signatures or specific types of substitutions (FDR < 0.1, Additional file 12: Figure S7a and b). The activity of the “AID/APOBEC-1” signature is associated with the genetic burden *CHEK2* (22q12.1; FDR = 0.0406), *HEATR3* (16q12.1; FDR = 0.0406) and *SMG6* (17p13.3; FDR = 0.0108; Additional file 12: Figure S7c). Whereas the activity of the “AID/APOBEC-2” signature is associated with the genetic burden of *DNAH11* (7p15.3; FDR = 0.0941), *HAP1* (17q21.2; FDR = 0.0941) and *HECTD4* (12q24.13; FDR = 0.0941; Additional file 12: Figure S7d).

### Multivariate analysis

To control for the confounding effects in the univariate analysis, we verified the associations aforementioned using multivariate regression, which accounts for the known clinical features of ESCC such as age, gender, stage. As results, we verified the associations between the somatic *TP53* mutations and the activities of the “NpCpG” signature (*P* = 2.21 × 10^− 7^; Fig. 3e), the “AID/APOBEC-2” signature, respectively (*P* = 2.00 × 10^− 4^; Fig. 3f). For *ZNF750*, we verified the associations with the activity of the “NpCpG” signature (*P* = 6.43 × 10^− 4^ for somatic status; Fig. 4e) and the “AID/APOBEC-2” signature (*P* = 3.47 × 10^− 2^ for somatic status and *P* = 1.26 × 10^− 6^ for genetic burden; Fig. 4f).And for *CDC27*, we verified its significant association with the activities of the “NpCpG” signature (*P* = 1.77 × 10^− 2^ for genetic burden and *P* = 6.71 × 10^− 3^ for somatic amplification, Fig. 5e) and the “AID/APOBEC-2” signature (*P* = 4.09 × 10^− 2^ for somatic amplification; Fig. 5f). It is also worth noting that the effects of these genes on the mutational signatures are highly consistent as revealed by both univariate and multivariate analyses.

We further looked into the genes which are significantly associated with somatic mutational signatures within each subtype. As results, we show that the associations between each gene and the activities of the mutational signatures varies considerably among the three subtypes of ESCC (Additional file 13: Figure S8).

## Discussion

The somatic landscape of the cancers are results of diverse mutational processes driven by both germline and somatic alterations in certain genes. Identification of the determinants of the mutational processes can inform the search for driver mutations and genes and thus enable a better understanding of esophagus carcinogenesis.

In this study, we report two new SMGs from a Chinese cohort of ESCC. *FAM90A1* is an unfavorable prognostic marker in endometrial cancer [34]. *TNRC6A* is an efficient biomarker for diagnosis and prognosis of non-small-cell lung cancer; and target of miR-30a [35]. All of the novel genes are functionally annotated in other tumor types. We also report some rare mutants with proved functions in ESCC. For instance, *CD7*, “a cell surface glycoprotein member of the immunoglobulin superfamily”, is up-regulated in Chinese ESCC patients and plays an essential role in T-cell and T-cell/B-cell interactions during early lymphoid development [36]. And *S100A2*, “a member of the S100 family of calcium-binding proteins”, is downregulated in a cohort of Chinese ESCC patients and related to the progression of ESCC [37].

Recent studies claim that both germline and somatic variants can influence the mutational processes in cancers [17–21]. In our analysis, we identified potential regulators of the mutational processes based on the evidences of associations at both germline and somatic levels. Our results highlight two genes, *ZNF750* and *CDC27*, of which the non-synonymous somatic mutations and germline genetic burden show consistent, significant effects on the activities of the mutational signatures. *ZNF750* functions as a regulator of epidermal cell differentiation by promoting differentiation genes while inhibiting progenitor factors. The tumor suppressing activity of *ZNF750* with significant prognostic power in multiple squamous cell carcinomas is confirmed by independent reports [38–42]. As for the other gene, Cell Division Cycle 27 is an activator of cAMP-dependent phosphorylation kinase activity [43]. The somatic copy-number amplification of *CDC27*, as an event of gain-of-function, is significantly associated with higher activity of the “NpCpG” signature whereas the high genetic burden in *CDC27* attenuates the activity of the “NpCpG” signature.

The driver mutations in cancer is often confounded by outnumbered passenger mutations, which can only be distinguished by systematic functional validation. We resort to the evidences from the germline genetics to prioritize the candidate somatic driver genes, and suggested determinants of the mutational processes in ESCC. While the clinical implication of *ZNF750* has been empirically proved by independent studies, *CDC27* is newly discovered for its effects on the mutational processes of ESCC. The method we described can be applied to many other cancer types to suggest candidate driver genes.

On the other hand, the discovery is based on a relatively small population, which means other subtle, independent mutational signatures in ESCC are yet to be discovered. The sample size can also hinder the discovery of other novel candidate genes due to the limitation in the statistical power. Plus, the current in silico analysis still does not fully reveal the underlying biology of the associations due to the lack of matched information such as mRNA transcription levels and complete pathological reports. The functional impacts of the candidate genes can be better addressed by further empirical validation in cell lines or mouse models.

Finally, our results suggest that the mutational processes in ESCC differ significantly between subpopulations of Chinese. Similar associations between the population-genetical background and the somatic mutations is previously reported in prostate cancer [44]. We also show that genes related to the GWAS risk loci can also influence the mutational processes in ESCC. Together these facts strongly suggest that germline variants help to better identify the driver genes of the somatic mutational processes in cancer.

## Conclusions

As conclusions, we report multiple factors that influence specific mutational processes in ESCC in a Chinese population, including the subpopulations, the SMGs, the genes related to the GWAS risk loci as well as exposure to alcohol and tobacco. We highlight *ZNF750* and *CDC27* as potential regulators of the mutational processes in ESCC by combining evidences at both germline and somatic levels. These findings inform the esophageal carcinogenesis and provide testable candidates for future functional studies.

## Methods

### Exome-sequencing data of ESCC

We obtained 9 ESCC samples from Zhongshan Hospital Xiamen University (ZHXU) in Xiamen, China. Tumor and matched normal tissues were collected after diagnosis and were stored at − 80 °C for subsequent DNA extraction. No patients were treated by chemotherapy or radiotherapy before the operation. All tumor tissues contained at least 80% malignant cells. We extracted DNA using QIAamp DNA Mini Kit (Qiagen) and prepared the whole-exome library using Agilent “SureSelect Human All Exon V5” following the standard protocol and perform 100 bp paired-end sequencing using Illumina HiSeq2000. All methods were performed in accordance with the relevant guidelines and regulations. This study is approved by the ethics committee/institutional review board (IRB) of ZHXU (approval no. 2017028) with written consent from all participants. The study complied with Declaration of Helsinki principles. The rest of 293 pairs of WES data sets were collected from published studies [20, 23–25]. All the sequencing data sets were based on paired ESCC and normal tissues from Chinese patients. The clinical information of the patients is either obtained by collected by ZHXU or previous studies (Additional file 14: Table S6).

### Somatic mutation analysis and genotyping

For the 302 WES data, we aligned the paired-end reads to human reference genome (hg19) using BWA (v0.7.12) [45]. The duplicated reads were removed using Picard Tools (v1.119; http://broadinstitute.github.io/picard/). We called somatic and germline variants using VarScan2 (v2.3.9) by controlling for mapping quality (≥ 30) and coverage (≥ 10) [46, 47]. The resulting SNVs and InDels were further filtered for alternate allele depth ≥ 10. We used GATK (v3.4.0) HaplotypeCaller for germline variants calling as well [48]. Then we chose the intersection of variants calls from GATK and VarScan2 for further analysis.

To determine the genotypes of the germline variants, we filtered the loci based on mapping quality (≥ 30), coverage of alternate allele (≥ 5) and the total coverage (≥ 20). We then determined the genotype based on alternate allele coverage rate (ACR) as the followings: (1) ACR ≤ 0.1, homozygote of the reference allele; (2) 0.2 ≤ ACR ≤ 0.8, heterozygote; (3) ACR ≥ 0.9, homozygote of the alternate allele. We intersected the resulting germline variants with the variants from the 1000 Genomes Project (TGP) phase 3 databases [49] then performed principal component analysis in a combined cohort of 103 CHB and 105 CHS from TGP and 302 ESCC samples.

### Somatic mutational signatures in ESCC

We retrieved highly conserved somatic mutational signatures using “pmsignature” [13]; and compared the results to the signatures in the COSMIC (http://cancer.sanger.ac.uk) [26] using CS measure.

### Somatic copy-number alterations

We used VarScan2 (v.2.3.9) to identify SCNAs with filtering conditions on the coverage (≥ 20), the base quality (≥ 20) and the mapping quality (≥ 20). The breakpoints were defined based on significant change of the ratio-of-depth between the tumor and the matched normal (*p* < 0.05 with Fisher’s Exact Test). We obtained the autosomal segments of copy-number changes using circular binary segmentation (CBS) and determined the somatic copy-number status of each segment by the log2-adjusted ratio-of-depth. A copy-number gain was defined if the corresponding value is above 0.5; and loss if the value is below − 0.5. JISTIC was used to find the significant regions and genes of SCNAs [50].

### Significantly mutated genes and pathways in ESCC

The somatic mutations were annotated using Oncotator (v1.5.1.0) [51]. Then we evaluated the enrichment of the non-synonymous somatic mutations (SMGs are FDR < 0.1) in each gene using MutSigCV (v1.4) [52]. We analyzed the pathways of genes that are either significantly frequently mutated (MutSigCV *p* < 0.05) and strongly associated with SCNAs using HotNet2 [53].

### Association analyses

We performed hierarchical clustering of the samples based on the Euclidean distances of the relative activities of the mutational signatures using Ward’s linkage method. We evaluated the associations between the subtypes and various risk factors (tobacco, alcohol exposure and populations), the total number of SNVs and the somatic statuses of genes. For the association analysis, we used χ^2^ tests for categorical variables and analysis of variance (ANOVA) for quantitative features.

We also evaluated the associations between mutational processes in ESCC and a set of genes of interests. We chose the total number of SNVs, the activities of the mutational signatures, the frequencies of each of the six types of base-substitutions (C > A, C > G, C > T, T > A, T > C and T > G) as proxies to the mutational processes. We determined the somatic statuses based on whether any non-synonymous somatic mutations present in a gene. We then compared the proxies between subsets defined by the somatic statuses of the genes of interests using Wilcoxon’s rank sum test.

To address the genetic burdens, we used the SNP-set Kernel Association Test (SKAT) to evaluate the association between the genetic burden of a given gene and the proxies of the somatic mutational processes [54]. For each gene, we chose all the exonic germline SNPs with no more than 50% missing calls in the population as the SNP-set. To avoid any confounding effects, we used the patients’ age, clinical stage and the first two principal components of the germline genotype (as surrogates for population variation) as covariates of the SKAT model. Thus, we tested 12 SMGs, 20 SCNA-related genes and 56 genes that are associated with known ESCC germline risk loci.

To better understand the association, we performed multivariate linear regression in ESCC samples and subgroup of ESCC:

$$ {\mathrm{S}\mathrm{ig}}_{ki}\sim {\beta}_0+{\upbeta}_1\times {\mathrm{G}}_{li}+{\upbeta}_2\times {\mathrm{S}}_{li}+{\upbeta}_3\times {\mathrm{gender}}_i+{\upbeta}_4\times {\mathrm{age}}_i+{\upbeta}_5\times {\mathrm{stage}}_i+{\upvarepsilon}_{kli} $$

where *ε*_*kli*_~*N* (0, *σ*^2^) is a Gaussian error term; Sig_*ki*_ corresponds to the *k*^*th*^ signature of the *i*^*th*^ sample; G_*li*_ corresponds to the genetic burden of the *l*^*th*^ gene in the *i*^*th*^ sample; S_*i*_ corresponds to the somatic mutational status of the *l*^*th*^ gene in the *i*^*th*^ sample; gender_*i*_ corresponds to the gender of the *i*^*th*^ sample; age_*i*_ corresponds to the age of the *i*^*th*^ sample;

$$ {\mathrm{G}}_{li}=\sum {W}_{lj}{g}_{li j} $$

$$ {g}_{lij}=\left\{\begin{array}{c}0,\kern0.5em homozygous\ reference\\ {}1,\kern0.5em heterozygote\kern2.25em \\ {}\begin{array}{cc}2,& homozygous\ alternate\end{array}\end{array}\right. $$

where *W*_*j*_ corresponds to the weight of the *j*^*th*^ SNP of the *l*^*th*^ gene, *g*_*lij*_ corresponds to the genotype of the *j*^*th*^ SNP of the *l*^*th*^ gene in the *i*^*th*^ sample;

$$ {W}_{lj}=1/\sqrt{MAF_{lj}\left(1-{MAF}_{lj}\right)} $$

where *MAF*_*lj*_ corresponds to the minimum allele frequency of the *j*^*th*^ SNP of the *l*^*th*^ gene;

$$ {\mathrm{S}}_{li}=\left\{\begin{array}{c}0,\kern0.5em {N}_{li}=0\\ {}1,\kern0.5em {N}_{li}>0\end{array}\right. $$

where *N*_*li*_ corresponds to the number of the non-synonymous mutation of the *l*^*th*^ gene in the *i*^*th*^ sample.

Thus, we looked into the genes which were significantly association with somatic mutational signatures in the previously individually correlation analysis.

## Additional files

Additional file 1: **Table S1.** Somatic mutations in 302 ESCC patients. (XLSX 785 kb)

Additional file 2: **Figure S1.** Significantly mutated genes in 302 ESCC. The samples are sorted by the counts of somatic mutations per megabase, with the synonymous and non-synonymous mutations shown in different colors. (top); the Significantly mutated genes (FDR < 0.1) are plotted for each patient carrying the mutations. Each row corresponds to a gene and each column a patient. The different types of mutations are color-coded (middle). (PDF 162 kb)

Additional file 3: **Table S2.** The functional annotations of in-frame mutations of *TNRC6A* and *FAM90A1*. (XLSX 10 kb)

Additional file 4: **Table S3.** The significant somatic copy-number alterations in 302 ESCC samples. The copy-number statuses, FDR and the mutated sample counts of peak regions are based on JISTIC. (XLSX 14 kb)

Additional file 5: **Table S4.** Genes of significant somatic copy-number alterations in ESCC. The copy-number GSCORE and FDR, the somatic copy-number statuses of each gene are based on JSTIC. A: amplification, D: deletion and N: neutral. (XLSX 34 kb)

Additional file 6: **Figure S2.** Significantly modified subnetworks in ESCC. The subnetworks are identified by HotNet2 from public protein-protein interactions databases of HINT+HI2012 (a), HPRD (b), iRefIndex (c) and MultiNet (d). The colored nodes represent the genes with different types of somatic alterations in ESCC, the sizes of the nodes correspond to the frequency of alteration in the population. All the subnetworks are identified with the minimum edge weight (δ), the minimum size of subnetwork (k) and the P less than 0.05. (PDF 158 kb)

Additional file 7: **Table S5.** The results of Gene Set Enrichment Analysis. (a) The subnetworks significantly enrich for KEGG pathways. (b) The subnetworks significantly enrich for Transcription factor targets. (XLSX 11 kb)

Additional file 8: **Figure S3.** Population stratification of 302 ESCC patients. Two hundred eight genotyped reference individuals are obtained from TGP including 103 CHB and 105 CHS. After filtering 20 outliers, the remaining 282 samples are classified into CHB and CHS at a threshold level of 0 for PC2. (PDF 165 kb)

Additional file 9: **Figure S4.** Comparison of total SNV counts and the frequencies of specific base substitutions with the somatic statuses of certain genes. (a) The total SNV counts are compared to the somatic statuses of *TP53*, *ZNF750*, *FAT1*, *FBXW7* and *PIK3CA*. (b) The frequencies of substitutions are compared to the somatic statuses of *TP53*. (c) The total SNV counts are compared to the somatic copy-number statuses of *MYC*, *CDC27* and *FHIT*. (d) The frequency of C > A substitution is compared to the somatic copy-number statuses of *CDC27*. FDR is based on the adjusted Wilcoxon rank-sum test *P* values. (PDF 418 kb)

Additional file 10: **Figure S5.** Correlation of the somatic events with the genetic burdens of the SMGs in ESCC. (a) The genetic burdens of *PTCH1* are associated with the activity of the “AID/APOBEC-2” signature. (b) The genetic burdens of *NOTCH1* are associated with the frequencies of C > G. (c) The genetic burdens of *TP53* and *PTCH1* are associated with the frequencies of T > C. FDR is based on the adjusted SKAT *P* values, in which the age, the clinical stage and ancestry are considered as covariates. (PDF 278 kb)

Additional file 11: **Figure S6.** Correlation of the somatic events with the genetic burdens of the SCNA-related genes in ESCC. The genetic burdens of *CDC27* are associated with the total number of SNVs (a) and the frequencies of C > G (b). The genetic burdens of *SLC6A3* are associated with the frequencies of T > G (c). The genetic burdens of *CDC27*, *RECQL*, *DGCR6* and *EGFR* are associated with the frequencies of T > C (d). FDR is based on the adjusted SKAT *P* values, in which the age, the clinical stage and ancestry are considered as covariates. (PDF 428 kb)

Additional file 12: **Figure S7** Correlation of the somatic events with the genetic burdens of the risk-associated genes in ESCC. The genetic burdens of *CHEK2* and *HECTD4* are associated with the frequencies of C > G substitution (a). The genetic burdens of *HEATR3* are associated with the frequencies of C > T substitution (b). The genetic burdens of *CHEK2*, *HEATR3* and *SMG6* are associated with the “AID/APOBEC-1” signature (c). The genetic burdens of *DNAH11*, *HAP1*, *HECTD4* and *HLA-DQA1* are associated with the “AID/APOBEC-2” signature (d). FDR is based on the adjusted SKAT *P* values, in which the age, the clinical stage and ancestry are considered as covariates. (PDF 407 kb)

Additional file 13: **Figure S8.** The effects of *TP53*, *ZNF750*, *CDC27* and other clinical features on the activity of the “NpCpG” signature and the “AID/APOBEC-2” signature in the subtypes of ESCC. The somatic status and the genetic burden of *TP53* influence the activities of the “NpCpG” signature (a) and the “AID/APOBEC-2” signature (b) in subtype 1 to 3, independent of other clinical features. And the somatic status and the genetic burden of *ZNF750* influence the activities of the “NpCpG” signature (c) and the “AID/APOBEC-2” signature (d) in subtype 1 to 3, independent of other clinical features. The somatic copy number amplification and the genetic burden of *CDC27* influence the activity of the “NpCpG” signature (e) and the “AID/APOBEC-2” signature (f) in subtype 1 to 3, independent of other clinical features. *P* values are based on multivariate linear regression analysis: *P < 0.05, **P < 0.01, ***P < 0.001. (PDF 892 kb)

Additional file 14: **Table S6.** Summary of the clinical features of the 302 ESCC patients included in this study. (XLSX 26 kb)

## Abbreviations

ACR: Alternate-allele Coverage Rate
ANOVA: Analysis of variance
CBS: Circular Binary Segmentation
CHB: Han Chinese in Beijing
CHS: Southern Han Chinese
COSMIC: Catalogue of Somatic Mutations in Cancer
CS: Cosine Similarity
EAC: Esophageal Adenocarcinoma
EC: Esophageal Cancer
ESCC: Esophageal Squamous Cell Carcinoma
InDels: Insertions and Deletions
PPI: Protein-protein Interaction
SCNAs: Somatic Copy-number Alterations
SKAT: SNP-set Kernel Association Test
SMGs: Significantly Mutated Genes
SNVs: Single Nucleotide Variants
TGP: The 1000 Genomes Project
WES: Whole-exome Sequencing
