# Supplementary figures and images for: Germline and somatic variations influence the somatic mutational signatures of esophageal squamous cell carcinomas in a Chinese population
Source: BMC Genomics. 2018 Jul 16;19:538. doi: 10.1186/s12864-018-4906-4 (PMC6048762; doi:10.1186/s12864-018-4906-4)

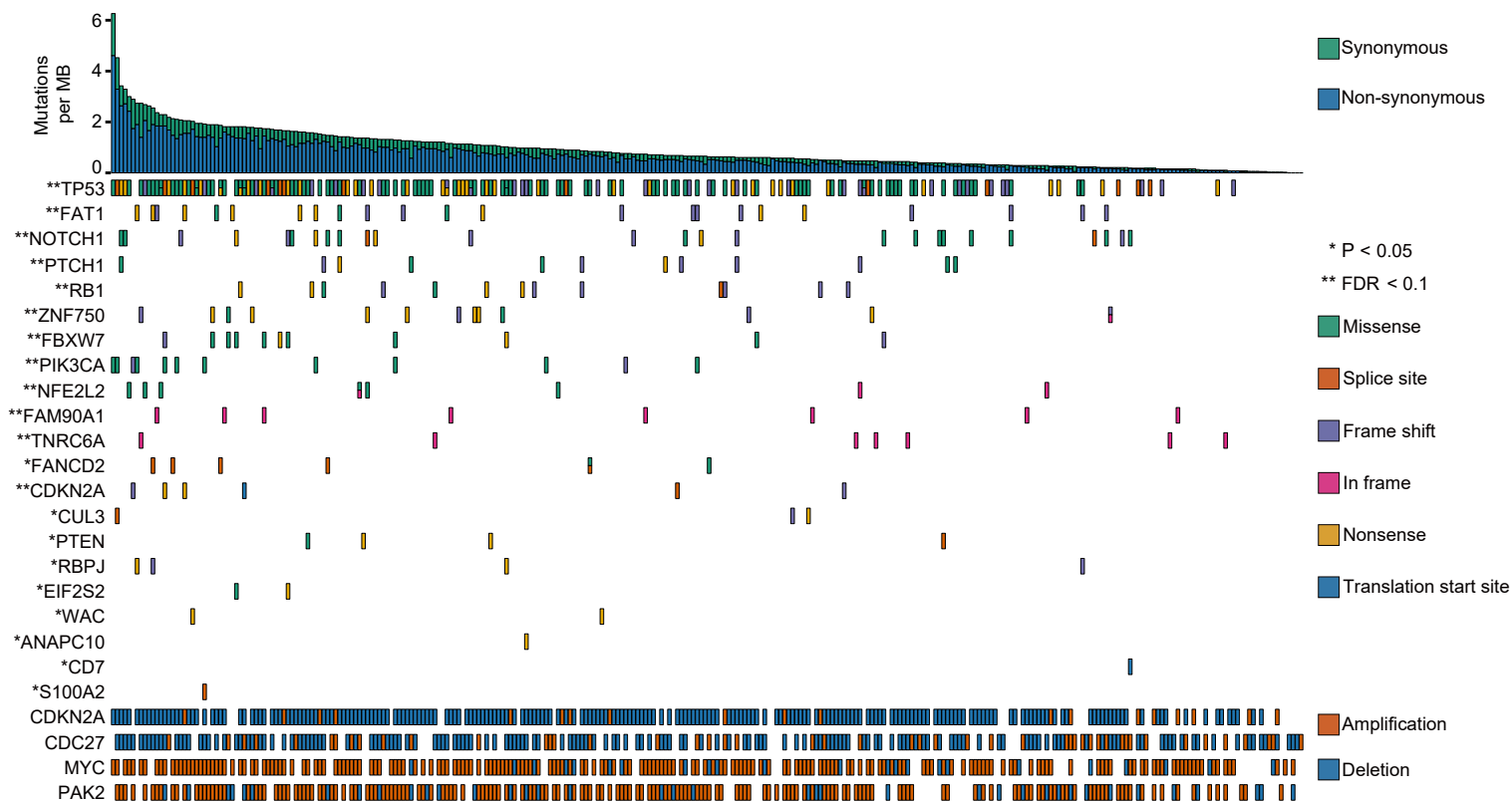

Figure S1

Supplement: Supplementary file 2 — Figure S1. Significantly mutated genes in 302 ESCC. The samples are sorted by the counts of somatic mutations per megabase, with the synonymous and non-synonymous mutations shown in different colors. (top); the Significantly mutated genes (FDR < 0.1) are plotted for each patient carrying the mutations. Each row corresponds to a gene and each column a patient. The different types of mutations are color-coded (middle). (PDF 162 kb) [file 12864_2018_4906_MOESM2_ESM.pdf]

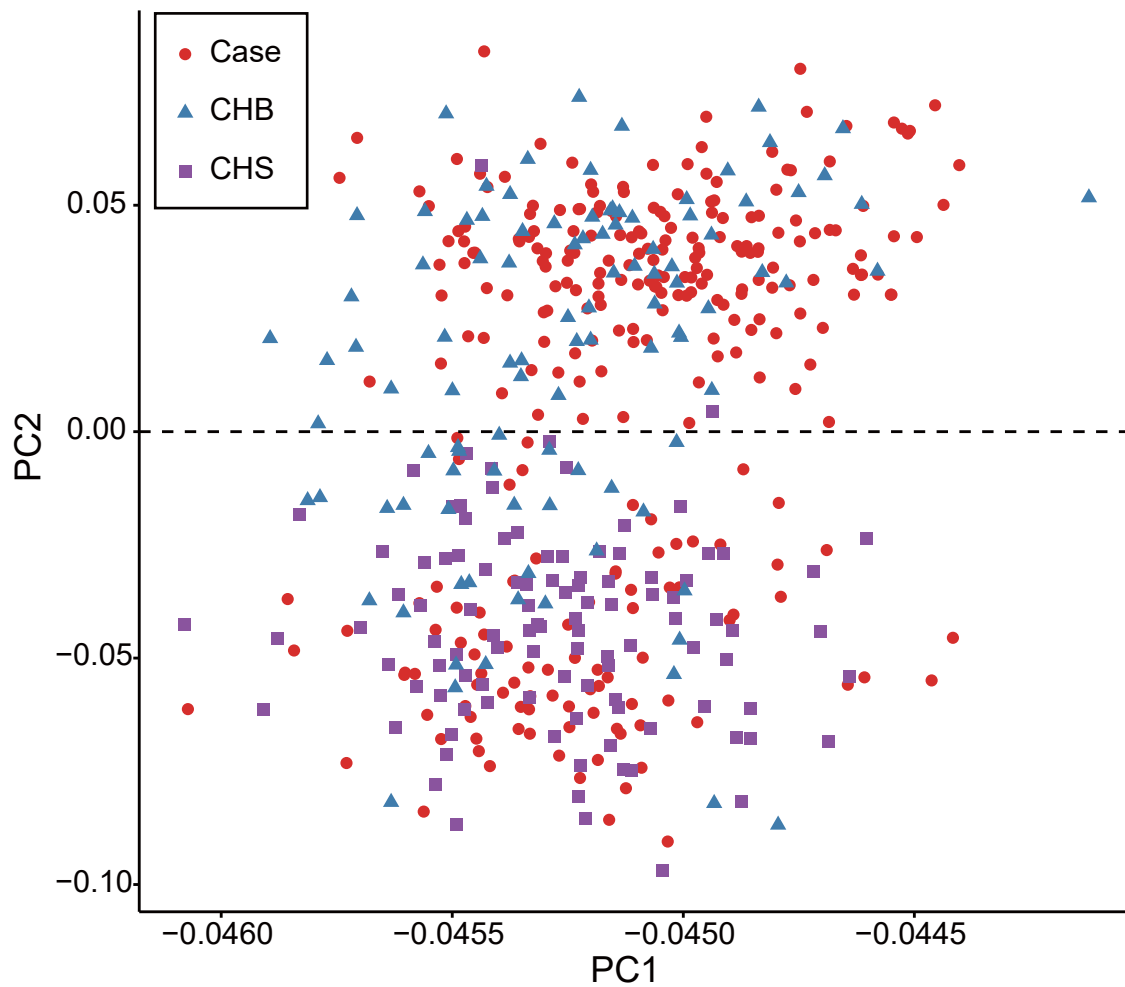

Figure S3

Supplement: Supplementary file 8 — Figure S3. Population stratification of 302 ESCC patients. Two hundred eight genotyped reference individuals are obtained from TGP including 103 CHB and 105 CHS. After filtering 20 outliers, the remaining 282 samples are classified into CHB and CHS at a threshold level of 0 for PC2. (PDF 165 kb) [file 12864_2018_4906_MOESM8_ESM.pdf]

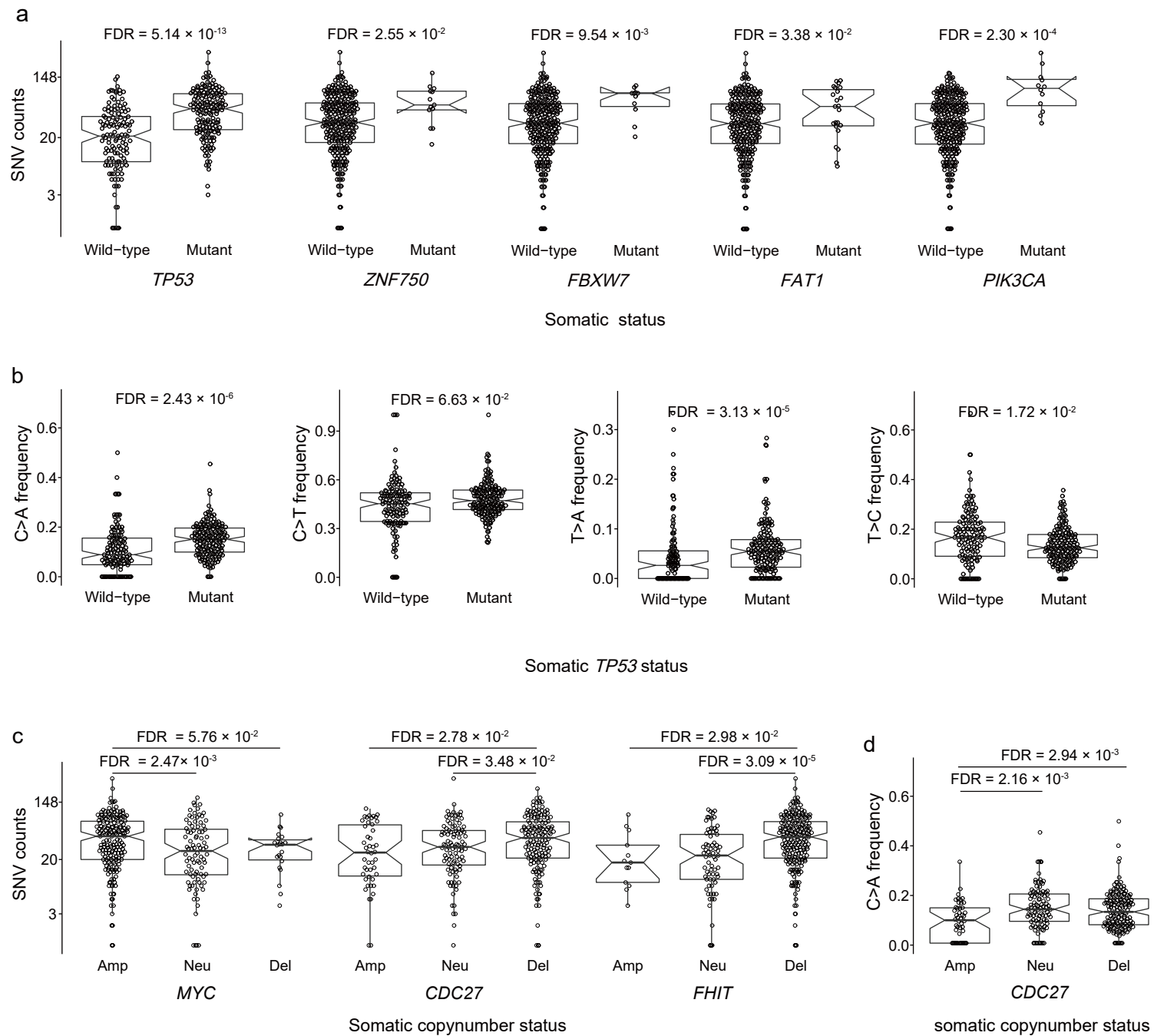

Figure S4

Supplement: Supplementary file 9 — Figure S4. Comparison of total SNV counts and the frequencies of specific base substitutions with the somatic statuses of certain genes. (a) The total SNV counts are compared to the somatic statuses of TP53, ZNF750, FAT1, FBXW7 and PIK3CA. (b) The frequencies of substitutions are compared to the somatic statuses of TP53. (c) The total SNV counts are compared to the somatic copy-number statuses of MYC, CDC27 and FHIT. (d) The frequency of C > A substitution is compared to the somatic copy-number statuses of CDC27. FDR is based on the adjusted Wilcoxon rank-sum test P values. (PDF 418 kb) [file 12864_2018_4906_MOESM9_ESM.pdf]

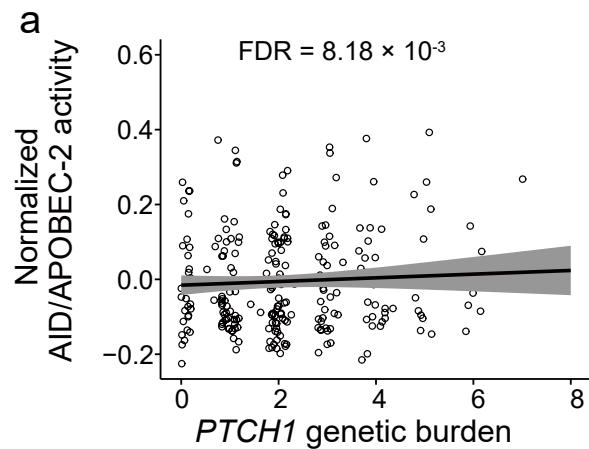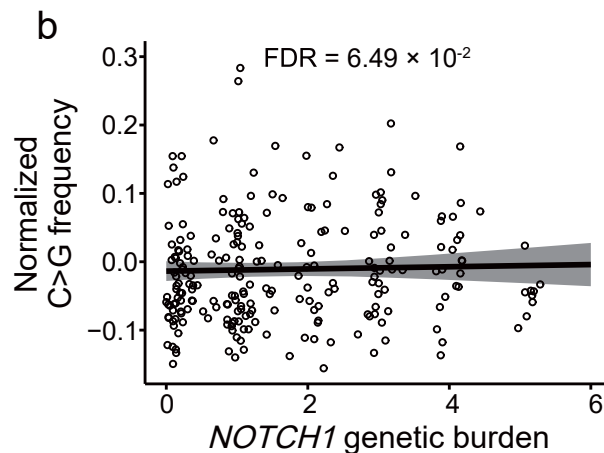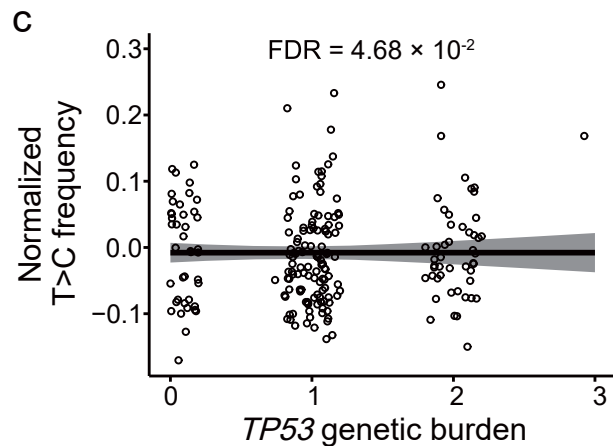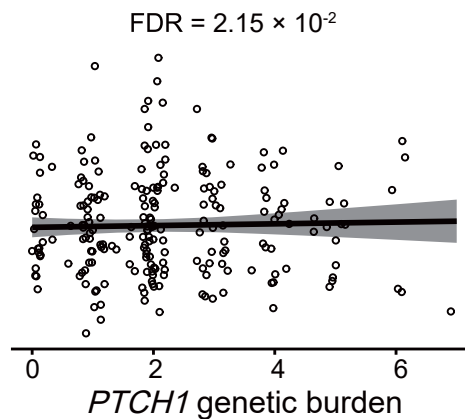

Figure S5

Supplement: Supplementary file 10 — Figure S5. Correlation of the somatic events with the genetic burdens of the SMGs in ESCC. (a) The genetic burdens of PTCH1 are associated with the activity of the “AID/APOBEC-2” signature. (b) The genetic burdens of NOTCH1 are associated with the frequencies of C > G. (c) The genetic burdens of TP53 and PTCH1 are associated with the frequencies of T > C. FDR is based on the adjusted SKAT P values, in which the age, the clinical stage and ancestry are considered as covariates. (PDF 278 kb) [file 12864_2018_4906_MOESM10_ESM.pdf]

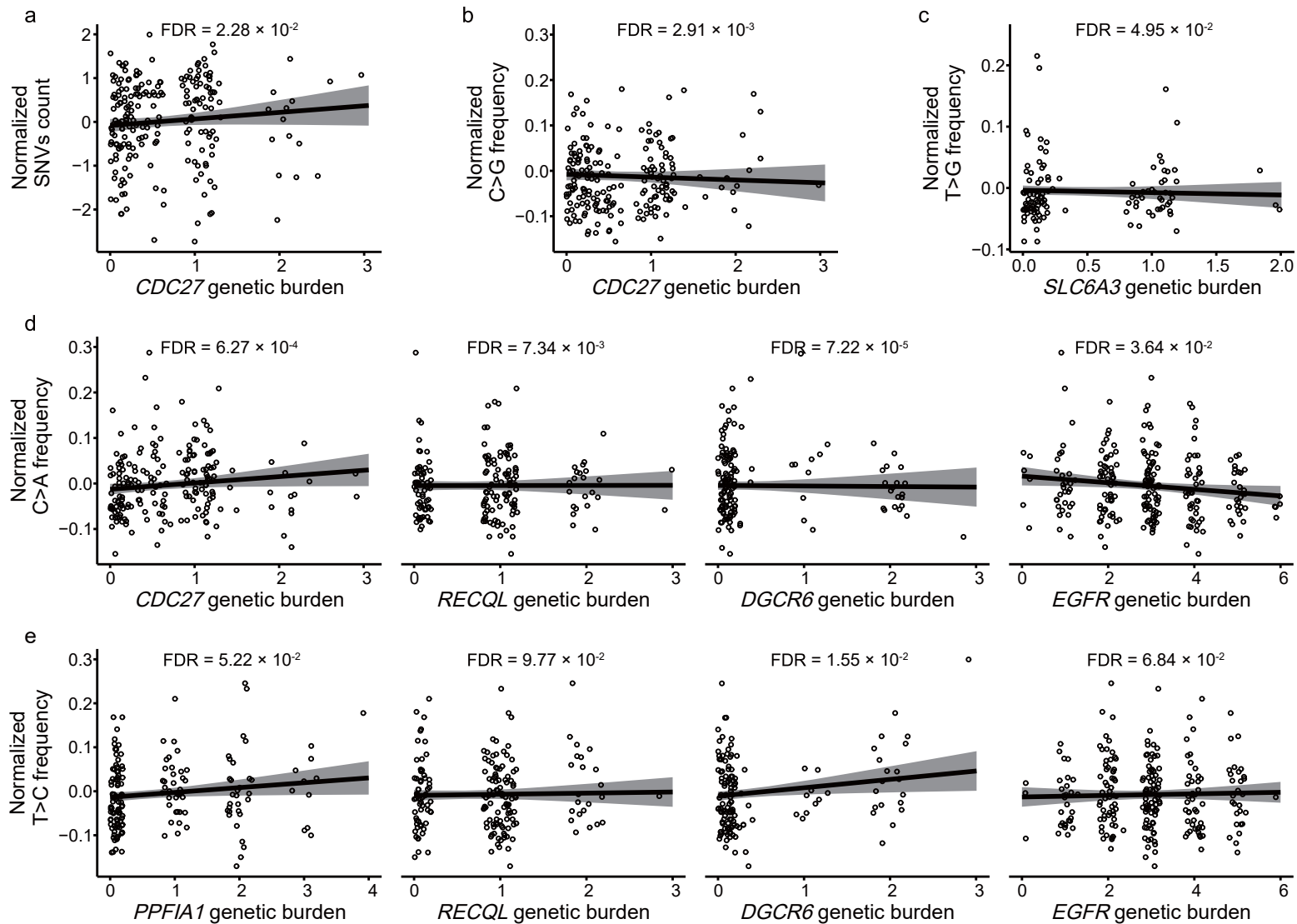

Figure S6

Supplement: Supplementary file 11 — Figure S6. Correlation of the somatic events with the genetic burdens of the SCNA-related genes in ESCC. The genetic burdens of CDC27 are associated with the total number of SNVs (a) and the frequencies of C > G (b). The genetic burdens of SLC6A3 are associated with the frequencies of T > G (c). The genetic burdens of CDC27, RECQL, DGCR6 and EGFR are associated with the frequencies of T > C (d). FDR is based on the adjusted SKAT P values, in which the age, the clinical stage and ancestry are considered as covariates. (PDF 428 kb) [file 12864_2018_4906_MOESM11_ESM.pdf]

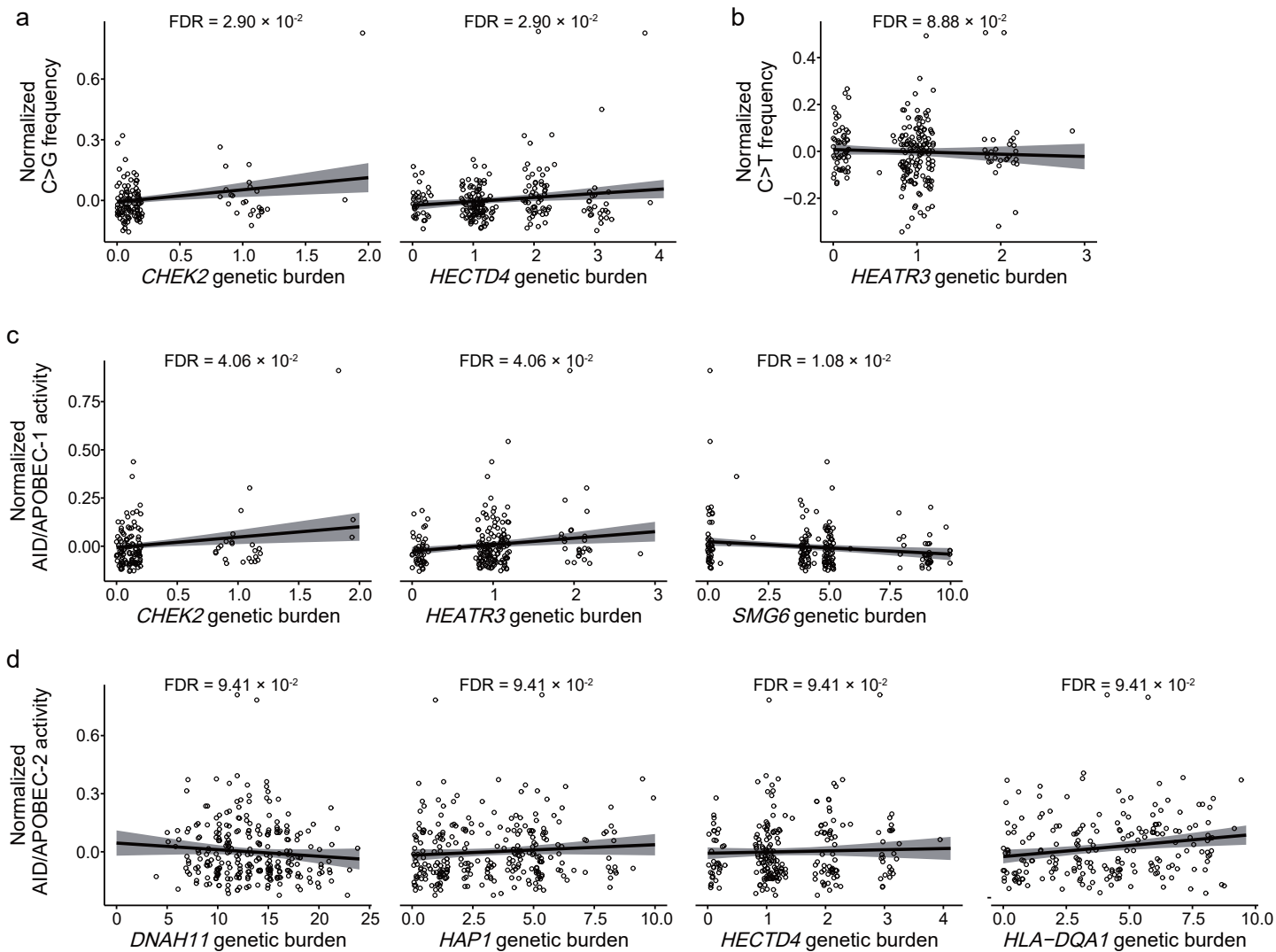

Figure S7

Supplement: Supplementary file 12 — Figure S7 Correlation of the somatic events with the genetic burdens of the risk-associated genes in ESCC. The genetic burdens of CHEK2 and HECTD4 are associated with the frequencies of C > G substitution (a). The genetic burdens of HEATR3 are associated with the frequencies of C > T substitution (b). The genetic burdens of CHEK2, HEATR3 and SMG6 are associated with the “AID/APOBEC-1” signature (c). The genetic burdens of DNAH11, HAP1, HECTD4 and HLA-DQA1 are associated with the “AID/APOBEC-2” signature (d). FDR is based on the adjusted SKAT P values, in which the age, the clinical stage and ancestry are considered as covariates. (PDF 407 kb) [file 12864_2018_4906_MOESM12_ESM.pdf]

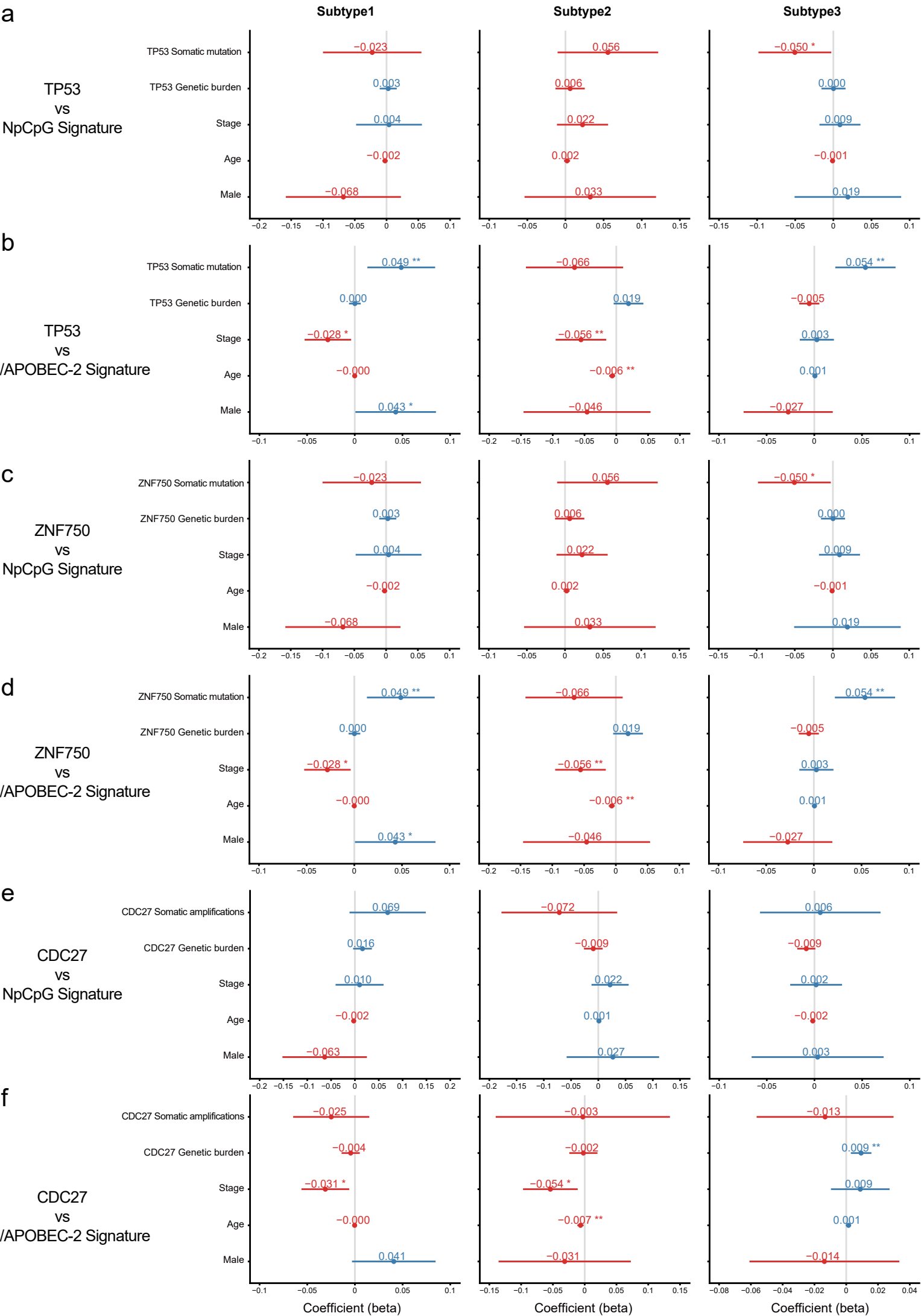

Figure S8

Supplement: Supplementary file 13 — Figure S8. The effects of TP53, ZNF750, CDC27 and other clinical features on the activity of the “NpCpG” signature and the “AID/APOBEC-2” signature in the subtypes of ESCC. The somatic status and the genetic burden of TP53 influence the activities of the “NpCpG” signature (a) and the “AID/APOBEC-2” signature (b) in subtype 1 to 3, independent of other clinical features. And the somatic status and the genetic burden of ZNF750 influence the activities of the “NpCpG” signature (c) and the “AID/APOBEC-2” signature (d) in subtype 1 to 3, independent of other clinical features. The somatic copy number amplification and the genetic burden of CDC27 influence the activity of the “NpCpG” signature (e) and the “AID/APOBEC-2” signature (f) in subtype 1 to 3, independent of other clinical features. P values are based on multivariate linear regression analysis: *P < 0.05, **P < 0.01, ***P < 0.001. (PDF 892 kb) [file 12864_2018_4906_MOESM13_ESM.pdf]
