# Supplementary material for: Germline and somatic variations influence the somatic mutational signatures of esophageal squamous cell carcinomas in a Chinese population
Source: BMC Genomics. 2018 Jul 16;19:538. doi: 10.1186/s12864-018-4906-4 (PMC6048762; doi:10.1186/s12864-018-4906-4)
Supplement: Supplementary file 6 — Figure S2. Significantly modified subnetworks in ESCC. The subnetworks are identified by HotNet2 from public protein-protein interactions databases of HINT+HI2012 (a), HPRD (b), iRefIndex (c) and MultiNet (d). The colored nodes represent the genes with different types of somatic alterations in ESCC, the sizes of the nodes correspond to the frequency of alteration in the population. All the subnetworks are identified with the minimum edge weight (δ), the minimum size of subnetwork (k) and the P less than 0.05. (PDF 158 kb) [file 12864_2018_4906_MOESM6_ESM.pdf]

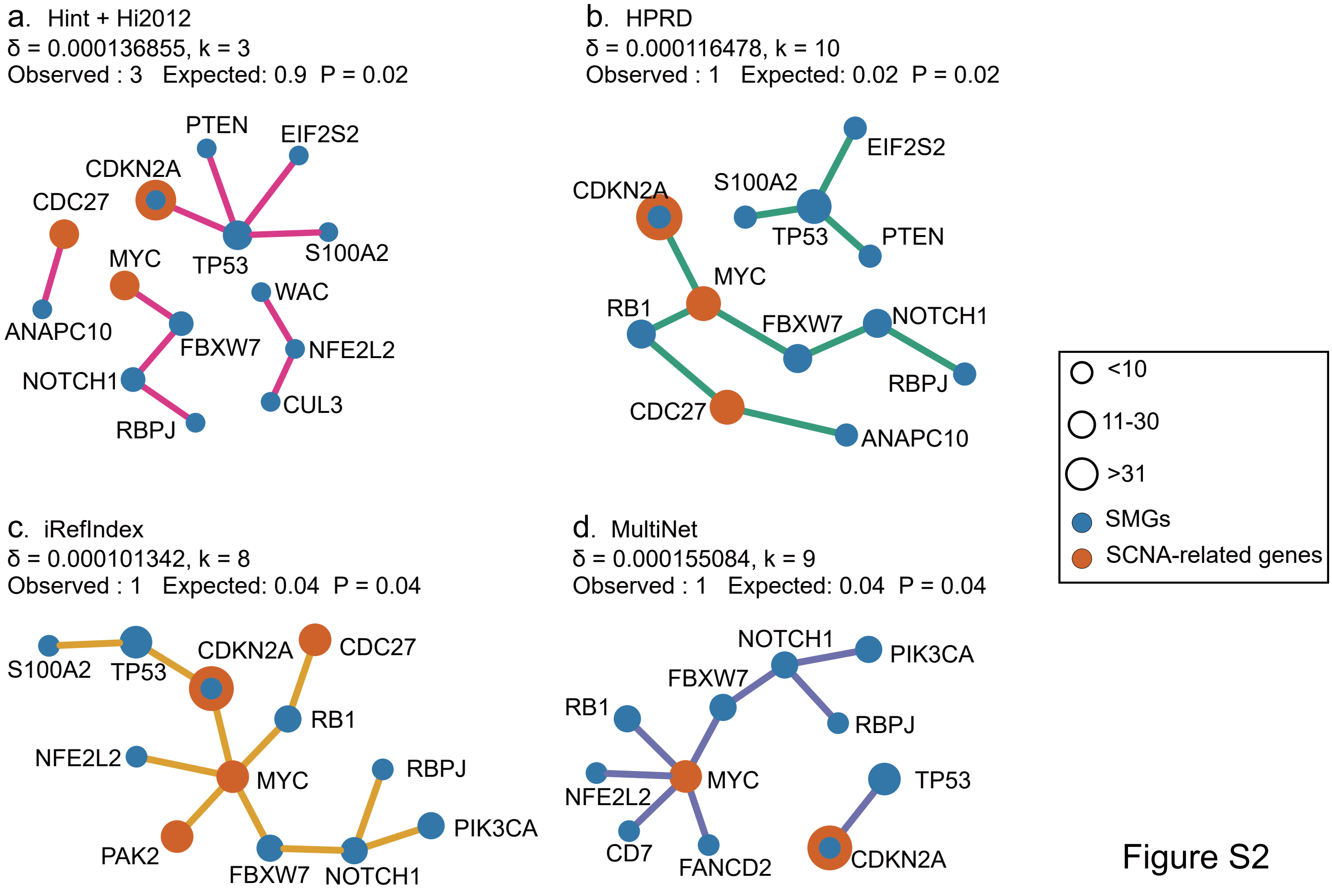
a. Hint + Hi2012
δ = 0.000136855, k = 3
Observed : 3 Expected: 0.9 P = 0.02
PTEN
EIF2S2
CDKN2A
CDC27
TP53
S100A2
MYC
WAC
ANAPC10
FBXW7
NFE2L2
NOTCH1
CUL3
RBPJ
b. HPRD
δ = 0.000116478, k = 10
Observed : 1 Expected: 0.02 P = 0.02
EIF2S2
S100A2
CDKN2A
TP53
PTEN
MYC
RB1
FBXW7
NOTCH1
RBPJ
CDC27
ANAPC10
<10
11-30
>31
SMGs
SCNA-related genes
c. iRefIndex
δ = 0.000101342, k = 8
Observed : 1 Expected: 0.04 P = 0.04
CDC27
S100A2
TP53
CDKN2A
RB1
NFE2L2
MYC
RBPJ
PIK3CA
PAK2
FBXW7
NOTCH1
d. MultiNet
δ = 0.000155084, k = 9
Observed : 1 Expected: 0.04 P = 0.04
NOTCH1
PIK3CA
FBXW7
RB1
RBPJ
MYC
TP53
NFE2L2
CD7
FANCD2
CDKN2A

Figure S2
